# Reduced HAV IgG Seropositivity Among Unvaccinated People Living with HIV: The Weak Shield

**DOI:** 10.3390/tropicalmed11060158

**Published:** 2026-06-11

**Authors:** Huan Xu, Sheng Huang, Haotian Huang, Xinrui Gao, Chunlin Chen, Guangyu Liang, Aili Lu, Xuwen Xu, Yuyuan Xu, Hao Wang, Xin Tao, Shaohang Cai

**Affiliations:** 1 Department of Infectious Diseases, Nanfang Hospital, Southern Medical University, Guangzhou 510515, China; xuhuansmu@163.com (H.X.); hush.01@163.com (S.H.); tanjs_0731@163.com (H.H.); gxrrrrrr2024@163.com (X.G.); arthur_scl@126.com (C.C.); lal03@yeah.net (A.L.); xuxuwen95@126.com (X.X.); xuyuyuan@sina.com (Y.X.); 2 State Key Laboratory of Organ Failure Research, Key Laboratory of Infectious Diseases Research in South China, Ministry of Education, Guangdong Provincial Key Laboratory of Viral Hepatitis Research, Guangdong Provincial Clinical Research Center for Viral Hepatitis, Guangdong Institute of Hepatology, Guangzhou 510515, China; 3 Department of Infectious Disease, The Islands Healthcare Complex—Macao Medical Center of Peking Union Medical College Hospital, Macao SAR, China; lgy0790@163.com; 4 The First School of Clinical Medicine, Nanfang Hospital, Southern Medical University, Guangzhou 510515, China

**Keywords:** HIV infection, HAV infection, immune function, immunoglobulin G, immunoglobulin M, vaccination

## Abstract

People living with HIV (PLWH) represent a high-risk population for hepatitis A virus (HAV) infection, with exposure risk equal to or higher than that of the general population, particularly within adult risk networks. Anti-HAV immunoglobulin G (IgG) serves as a neutralizing antibody and is considered a key marker of protective immunity against HAV infection. However, the serologic profile of anti-HAV IgG and IgM among unvaccinated PLWH remains insufficiently characterized, especially in South China. A total of 1232 unvaccinated adults were enrolled in the study, including 800 PLWH and 432 HIV- negative controls, to evaluate the serological markers of HAV immunity. Serum anti-HAV IgG and immunoglobulin M (IgM) were measured using enzyme-linked immunosorbent assays, and demographic, immunological, and biochemical data were collected. We observed that PLWH had significantly lower anti-HAV IgG concentrations (0.27 ± 0.16 vs. 0.31 ± 0.14 ng/mL, *p* < 0.001) and a lower IgG seropositivity rate (22.5% vs. 35.6%, *p* < 0.001) compared with HIV-negative controls, whereas no differences were found in IgM levels or positivity between the two groups. Multivariable logistic regression identified HIV infection (OR = 0.599, 95% CI 0.413–0.869, *p* = 0.007) and age (OR = 1.019, 95% CI 1.007–1.031, *p* = 0.002) as independent factors associated with IgG seropositivity. Among PLWH, those who were IgG-positive tended to be older (*p* = 0.003) and had higher serum globulin levels (*p* < 0.001), whereas IgM positivity was linked to younger age (*p* < 0.001) and a higher CD4/CD8 ratio (*p* = 0.030). Age-stratified analyses further revealed that IgG seroprevalence increased with age, while IgM positivity showed a declining trend. These findings indicate a considerable immunity gap in a population at elevated risk of HAV infection and support the need for targeted serologic screening and vaccination strategies among PLWH.

## 1. Introduction

HAV infection remains a major global public health burden, causing tens of millions of new cases and substantial mortality each year, with disproportionate impact in settings where sanitation is limited [1]. HAV is predominantly transmitted via the fecal–oral route, and its environmental stability facilitates outbreaks associated with contaminated food and water [2,3]. In regions with improving hygiene, HAV transmission patterns have shifted; outbreaks increasingly concentrate in specific adult risk networks. Importantly, among men who have sex with men (MSM), sexual transmission via oral–anal contact can support effective chains of transmission, resulting in prolonged community outbreaks, as observed in Europe, Australia, and the Americas [4]. Although HAV infection is typically self-limited in immunocompetent adults, the risk of severe hepatitis and acute liver failure rises in older adults and in those with chronic liver disease or compromise immune systems [3,5]. Thus, HAV continues to represent a meaningful threat to adults with defined comorbidities or behavioral risk profiles even when vaccinated.

China’s epidemiology of HAV has experienced a profound transition. Following the integration of the hepatitis A vaccination into the National Immunization Program (NIP) in 2008, childhood coverage increased dramatically, leading to a significant decline in overall incidence [6]. Childhood vaccination is provided free of charge, and national coverage has remained above 83% in recent years [7]. In contrast, hepatitis A vaccination for adults is voluntary and self-funded rather than mandatory. In the meantime, this policy effectively created two immunologically distinct cohorts: individuals born after policy implementation are primarily vaccine-protected, whereas a large proportion of adults born earlier depend on naturally acquired immunity. Crucially, national serosurveys indicate that the 1988–2004 birth cohort (ages 19–35 during the study period) has a low anti-HAV IgG seroprevalence (<50%), reflecting both low vaccination uptake and reduced natural infection due to improved sanitation, rendering them a susceptible population [6].

Between 2005 and 2023, a total of 605,509 hepatitis A cases were reported in China, corresponding to an average annual incidence of 2.32 per 100,000 population. The disease burden varied by demographic factors and geographic region. Incidence was higher in males (2.85 per 100,000) than in females (1.81 per 100,000). Although overall HAV incidence declined substantially over the study period, the decrease was less pronounced among individuals aged ≥65 years, and their relative case proportion increased [8]. These national data offer a benchmark for interpreting regional serological findings and confirm that China has transitioned to low-to-moderate HAV endemicity. Nevertheless, studies specifically examining the incidence or seroprevalence of HAV among PLWH in China remain scarce, highlighting a notable gap in the research that the present study seeks to address.

In immunocompetent hosts, natural HAV infection generally induces durable, likely lifelong anti-HAV IgG responses [9]. However, the durability of HAV immunity in PLWH requires critical re-examination. HIV infection is associated with broad immune dysfunction, including disrupted B-cell homeostasis, the depletion of memory B-cell compartments, and impaired germinal center function, mechanisms that may weaken the generation and long-term maintenance of humoral immune memory [10]. These defects are reflected in well-documented reductions in vaccine responsiveness and protective durability to other antigens, including hepatitis B, COVID-19, and influenza [11,12,13].

According to previous studies [14], PLWH experience a significantly longer median time to the complete resolution of acute hepatitis A compared to their HIV-negative counterparts. Concurrently, the incidence of a prolonged disease course, defined as the persistence of symptoms and abnormal liver function for more than eight weeks, is also markedly higher in this population, yet they exhibited less severe hepatocellular injury (lower ALT peaks), indicating a blunted immune response despite greater viral persistence [14]. In PLWH co-infected with hepatitis B (HBV) or hepatitis C (HCV), disease progresses faster, immune responses change, and the risk of poor outcomes rises markedly. This well-established pattern offers a key reference for hypothesizing how HAV co-infection might behave in the same population. Existing evidence suggests that individuals with HIV co-infection, particularly those with severe immunosuppression (low CD4^+^ T cell counts) or pre-existing chronic liver disease (e.g., HCV co-infection), may carry an increased risk of progressing to severe hepatitis, acute liver failure, or even mortality following HAV infection [15,16,17]. Clinically, these mechanisms may manifest as elevated liver enzyme levels (ALT/AST), more marked jaundice, and the slower recovery of liver function, with some patients experiencing a protracted or atypical clinical course [16,18]. These findings imply that HAV-specific immune control is weakened in PLWH. Consequently, a prior infection does not necessarily equate to protective immunity in this group.

Consequently, a key clinical question follows: among unvaccinated adults who likely acquired HAV immunity through natural infection prior to widespread vaccination, do PLWH sustain comparable levels of protective anti-HAV IgG, or does HIV-associated immune dysregulation lead to defects in humoral memory? This question is especially urgent for MSM living with HIV, who face a dual risk which includes heightened exposure due to sexual transmission networks and the potential loss of protection due to their immunocompromised status, as suggested by HAV outbreaks in MSM populations globally [19].

Therefore, we conducted a study to compare anti-HAV IgG seropositivity and titers between unvaccinated adult PLWH and HIV-negative controls, and to evaluate clinical and immunologic correlates of HAV antibody status within PLWH, including age, CD4^+^ counts, CD4/CD8 ratio, and ART status. Our findings aim to inform targeted screening and vaccination strategies for this high-risk population.

## 2. Materials and Methods

### 2.1. Study Design, Setting, and Participants

**Figure 1:**
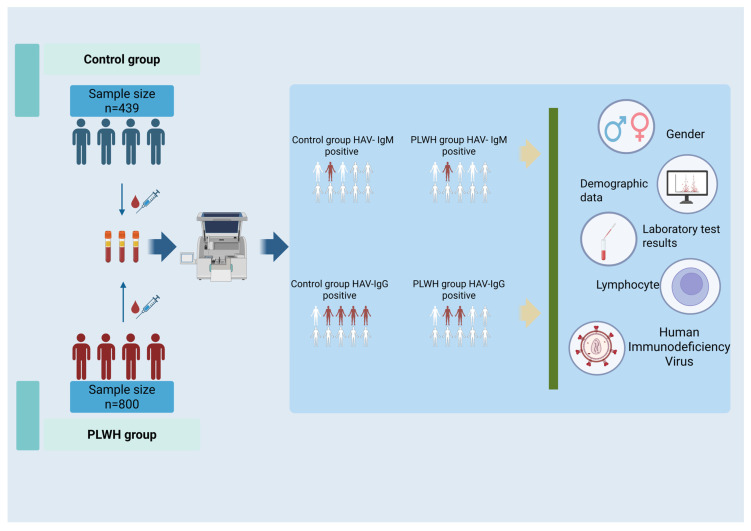
Study design and group comparisons. The schematic illustrates the study population and analytical framework. The figure was created with BioRender.

This study was designed as a hospital-based, single-center, cross-sectional observational study conducted at the Nanfang Hospital, Southern Medical University, Guangzhou, China. PLWH were consecutively recruited from the Department of Infectious Diseases, Nanfang Hospital between 1 March 2022 and 31 March 2025. HIV-negative controls were recruited from individuals undergoing routine health examinations at the Health Management Center of the same hospital during the same period. A schematic overview of the study is provided (Figure 1). Demographic and clinical characteristics were collected.

The study population consisted of adults aged ≥18 years, including PLWH and HIV-negative controls, who had available serum samples for anti-HAV antibody testing during the study period. A consecutive sampling strategy was adopted to recruit all eligible participants during the enrollment period. Eligibility criteria were as follows: Inclusion criteria: (1) Age ≥ 18 years; (2) Availability of serum samples for anti-HAV testing; (3) Provision of written informed consent. For the PLWH group, confirmed HIV-1 infection was additionally required. Exclusion criteria: (1) history of hepatitis A vaccination; (2) insufficient serum samples for anti-HAV testing; and (3) missing key clinical or laboratory data required for analysis.

This study received approval by the Institutional Ethics Committee of Nanfang Hospital (research identifier: NFEC-2021-448) and was conducted in accordance with the principles of the 1964 Declaration of Helsinki and its subsequent revisions. Written informed consent was obtained from each patient, who agreed to adhere to the study protocol and permitted the anonymous publication of their medical record details.

### 2.2. Clinical Data Extraction

Peripheral blood samples were obtained from all participants. HIV screening was performed with certified immunoassays and confirmed by Western blot analysis. Standard-of-care testing included CD4^+^ and CD8^+^ T cell counts, plasma viral load, and serology for HBV and syphilis (RPR-Rapid Plasma Reagin Circle Card Test). Hematologic indices and biochemistry parameters were retrieved, including white blood cells (WBC), platelets (PLT), lymphocyte (LYM) counts, baseline CD4^+^/CD8^+^ indices, anti-HAV IgG/IgM, HBsAg, and RPR. Serum samples were processed and stored at −80 °C until analysis. Data were collected from the electronic medical record system. CBC was measured using a Sysmex SE-9000 automated hematology analyzer (Sysmex Corporation, Kobe, Japan), including WBC, LYM, and PLT counts. Liver function tests were performed using an Olympus AU-5400 analyzer (Olympus Corporation, Tokyo, Japan), measuring ALT, AST, total bilirubin (TBIL), direct bilirubin (DBIL), and albumin (ALB).

### 2.3. Anti-HAV IgG and IgM Antibody Detection Assay

Anti-HAV IgG antibodies were measured using a double-antibody sandwich ELISA kit (MM-62610H1, MEIMIAN, Yancheng, China) according to the manufacturer’s instructions. Anti-human IgG (Fc-specific)-coated plates were blocked with 5% BSA, and diluted serum samples (1:100) were incubated at 37 °C for 60 min. After washing, HAV antigen and HRP-conjugated anti-HAV IgG antibodies were sequentially added. Color was developed using 100 μL TMB and absorbance was measured at 450 nm. Each sample was tested in triplicate and averaged. IgG concentrations were semi-quantitatively determined using a standard curve. Samples were considered positive at concentrations ≥ 2.5 ng/mL. Anti-HAV IgM antibodies were detected using a direct ELISA kit (MM-63114H1, MEIMIAN, Yancheng, China) following the manufacturer’s protocol. Serum samples (1:10 dilution) were incubated on antigen-coated plates at 37 °C for 60 min, followed by HRP-conjugated anti-human IgM. After TMB development, absorbance was read at 450 nm. Each sample was analyzed in triplicate. Positivity was defined as OD ≥ (mean negative control + 2 SD). Positive and negative controls provided by the manufacturer were included in each run, and results were considered valid only when quality control criteria were met. All testing was performed in Nanfang Hospital.

### 2.4. Statistical Analysis

Analyses were conducted in SPSS v25.0 (SPSS Inc., Chicago, IL, USA). For continuous variables, data are shown as mean ± SD or median (IQR), and between-group differences were assessed using a Student’s *t*-test or the Mann–Whitney U test, depending on normality. Categorical variables, reported as *n* (%), were compared via χ^2^ or Fisher’s exact tests. Multivariable analysis results were reported as ORs with 95% CIs. All variables assessed in the univariable analyses were subsequently entered into the multivariable logistic regression models without applying a predefined *p*-value cutoff in order to minimize potential selection bias and to comprehensively evaluate their independent associations after mutual adjustment. Correlations among continuous variables were assessed using Pearson or Spearman coefficients and visualized using correlation matrices. Two-sided *p* < 0.05 was considered statistically significant. Schematics were created with BioRender. Statistical charts and visualizations (including multivariable and correlation analyses) were generated using GraphPad Prism (v8.0) and R (v4.3.2).

## 3. Results

### 3.1. Clinical and Demographic Profiles of Two Groups

A total of 1232 participants were included in the final analysis, comprising 800 PLWH and 432 HIV-negative controls. Clinical and demographic profiles were shown in Table 1. The two groups were comparable in age (*p* = 0.151) and body mass index (*p* = 0.457) with the overall comparability of the two groups.

### 3.2. HAV Serologic Status Among Two Groups

**Figure 2:**
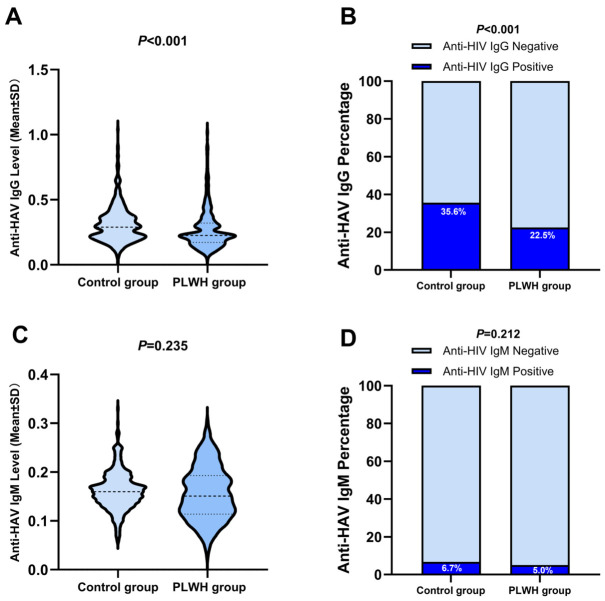
A comparison of IgM and IgG titers and positivity rates between the control group and the PLWH group. (**A**) Serum levels of HAV-specific IgG were significantly higher in the control group than in the PLWH group (0.31 ± 0.14 vs. 0.27 ± 0.16, *p* < 0.001). (**B**) The proportion of people with seropositivity IgG against HAV in the control group was higher than in the PLWH group (35.60% vs. 22.50%, *p* < 0.001). (**C**) Serum levels of HAV-specific IgM were similar in both groups (0.15 ± 0.05 vs. 0.16 ± 0.05, *p* = 0.235). (**D**) The proportion of people with IgM positivity was similar in both groups (6.70% vs. 5.00%, *p* = 0.212).

When serological profiles were assessed, distinct between-group differences were observed. The mean concentration of HAV-specific IgG was significantly lower in PLWH compared with the controls (0.27 ± 0.16 ng/mL vs. 0.31 ± 0.14 ng/mL, *p* < 0.001, Figure 2A). Correspondingly, the proportion of individuals seropositive for anti-HAV IgG was markedly reduced in PLWH (22.5% vs. 35.6%, *p* < 0.001, Figure 2B).

In contrast, anti-HAV IgM showed no significant between the PLWH group and the control group. The mean anti-HAV IgM titer was comparable between PLWH and controls (0.16 ± 0.05 S/CO vs. 0.15 ± 0.05 S/CO, *p* = 0.235, Figure 2C). Similarly, the prevalence of anti-HAV IgM seropositivity did not differ significantly (5.0% in PLWH vs. 6.7% in controls, *p* = 0.212, Figure 2D).

### 3.3. Multivariable Logistic Regression for IgM and IgG Seropositivity

**Figure 3:**
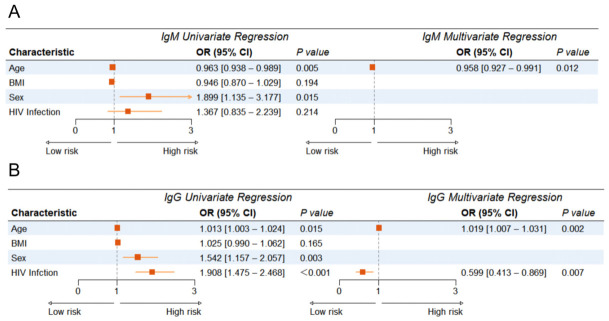
Multivariate analyses of factors associated with HAV IgM and IgG seropositivity among the control group and the PLWH group. (**A**) depicts the multivariate analysis for serum anti-HAV IgM, while (**B**) depicts the corresponding analysis for serum anti-HAV IgG.

A multivariable logistic regression model was used to analyze risk factors associated with HAV markers. For IgM positivity, age is the only independent factor associated with IgM positivity among all participants (OR = 0.958, 95% CI 0.927–0.991, *p* = 0.012, Figure 3A). For IgG positivity, the analysis identified two independent determinants including age (OR = 1.019, 95% CI 1.007–1.031, *p* = 0.002, Figure 3B) and HIV infection status (OR = 0.599, 95% CI 0.413–0.869, *p* = 0.007, Figure 3B), providing evidence that HIV infection is independently associated with a lower probability of anti-HAV IgG seropositivity after accounting for age and other candidate variables.

### 3.4. Heterogeneity of HAV Immune Responses Within PLWH Cohort

We further analyzed the demographic and clinical characteristics of PLWH with distinct HAV immunological status. Subgroup analyses were conducted according to HAV serologic profiles in the PLWH group. Among PLWH, individuals with anti-HAV IgM positivity were significantly younger than IgM-negative individuals (31.23 ± 6.84 years vs. 36.19 ± 12.06 years, *p* < 0.001). In addition, IgM-positive participants exhibited a higher current CD4/CD8 ratio (0.96 ± 0.52 vs. 0.77 ± 0.90, *p* = 0.030), as shown in Table 2.

As shown in Table 3, IgG-positive individuals were older than IgG-negative individuals (38.49 ± 13.58 years vs. 35.20 ± 11.27 years, *p* = 0.003). In addition, serum globulin levels were significantly higher in IgG-positive PLWH (30.01 ± 8.04 g/L vs. 27.34 ± 4.57 g/L, *p* < 0.001).

### 3.5. Multivariable Analysis of HAV Immune Status in PLWH

**Figure 4:**
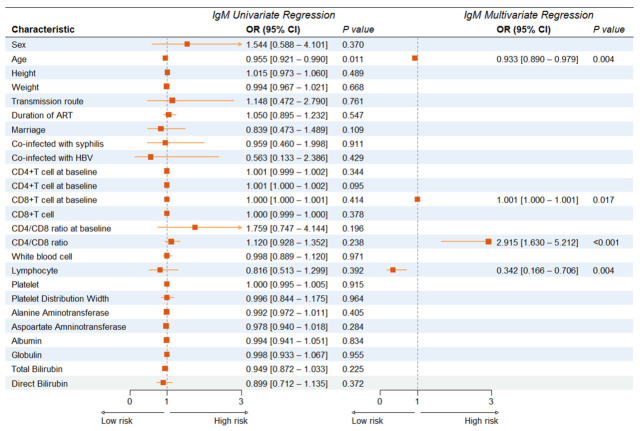
Multivariate analysis of IgM positivity in PLWH group. Multivariate analysis identified age, baseline CD8^+^ T cell count, CD4/CD8 ratio, and lymphocyte count as independent correlates. The results were as follows: Age (OR 0.933, 95% CI 0.890–0.979, *p* = 0.004), baseline CD8^+^ T cell count (OR 1.001, 95% CI 1.000–1.001, *p* = 0.017), CD4/CD8 ratio (OR 2.915, 95% CI 1.630–5.212, *p* < 0.001), and lymphocyte count (OR 0.342, 95% CI 0.166–0.706, *p* = 0.004).

We further performed PLWH-restricted multivariable analyses to evaluate independent factors correlated with HAV serum markers. For IgM positivity, four variables independently associated with IgM positivity among PLWH including age (OR = 0.993, 95% CI 0.890–0.979, *p* = 0.004), baseline CD8^+^ T cell count (OR = 1.001, 95% CI 1.000–1.001, *p* = 0.017), CD4/CD8 ratio (OR = 2.915, 95% CI 1.630–5.212, *p* < 0.001), and lymphocyte count (OR = 0.342, 95% CI 0.166–0.706, *p* = 0.004) (Figure 4).

**Figure 5:**
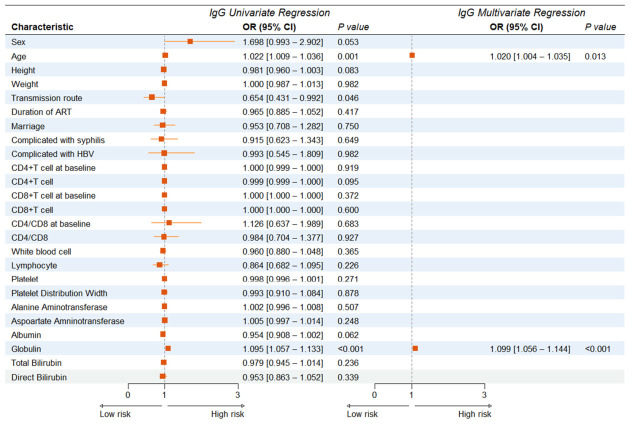
Forest plot of univariate and multivariate regression analysis for the association of various characteristics with IgG positivity rate in the PLWH group. Multivariate analysis identified age and globulin as independent correlates. The results were as follows: Age (OR 1.020, 95% CI 1.004–1.035, *p* = 0.013) and globulin (OR 1.099, 95% CI 1.056–1.144, *p* < 0.001).

For IgG positivity, multivariable analysis indicated that age (OR = 1.020, 95% CI 1.004–1.035, *p* = 0.013) and serum globulin (OR = 1.099, 95% CI 1.056–1.144, *p* < 0.001) were independently associated with IgG positivity (Figure 5).

### 3.6. Correlation Analysis of Anti-HAV IgG/IgM in PLWH

**Figure 6:**
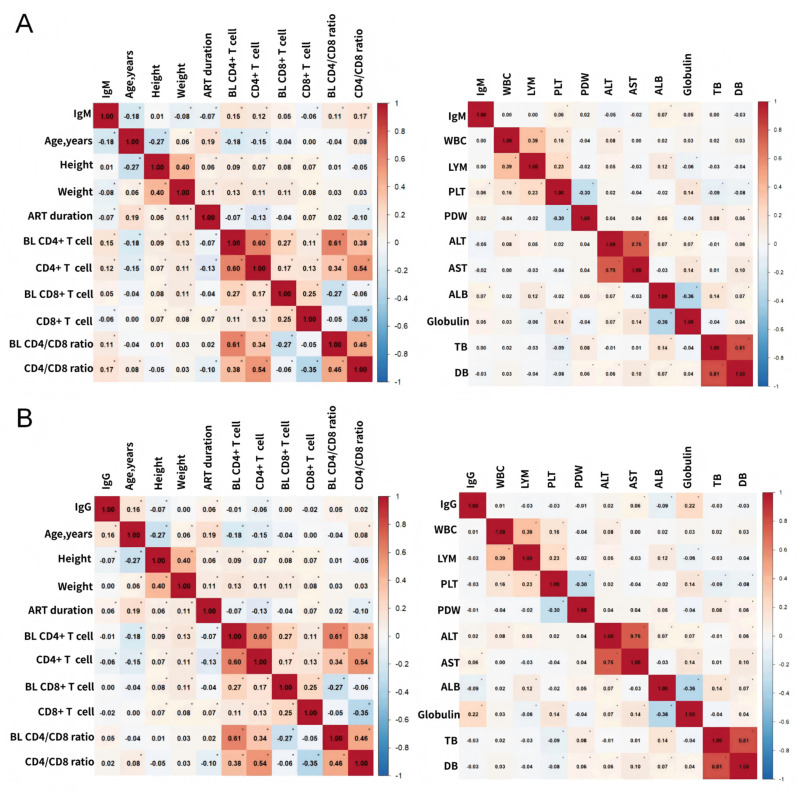
Correlation matrix analysis showing the associations of various characteristics with IgG and IgM titers in the HAV-co-infected PLWH group. Panel (**A**) corresponds to IgG titers, and Panel (**B**) to IgM titers. Abbreviations: IgM: Immunoglobin M; IgG: Immunoglobin G; BL-CD4^+^ T cell: CD4^+^ T cell at baseline; BL-CD8^+^ T cell: CD8^+^ T cell at baseline; BL CD4/CD8 ratio: CD4^+^ T cell/CD8^+^ T cell at baseline; WBC: White Blood Cell Count; LYM: Lymphocyte Count; PLT: Platelets; PDW: Platelet Distribution Width; ALT: Alanine Aminotransferase; AST: Aspartate Aminotransferase; ALB: Albumin; TB: Total Bilirubin; DB: Direct Bilirubin. The ‘*’ represents a conventional notation for statistical significance (*p* < 0.05).

We performed correlation analyses to evaluate the relationships between anti-HAV IgM/IgG antibody titers and a panel of demographic, immunological, and clinical laboratory parameters. The complete correlation matrix is presented in Figure 6.

Anti-HAV IgM titers demonstrated limited associations with the measured variables. A slight negative correlation was observed with age (r = −0.18). Positive correlations of modest magnitude were noted with baseline CD4^+^ T cell count (r = 0.15) and current CD4^+^ T cell count (r = 0.12). IgM levels showed no meaningful correlation with body weight (r = −0.08), current CD8^+^ T cell count (r = −0.06), or duration of antiretroviral therapy (ART, r = −0.07).

In contrast, anti-HAV IgG titers exhibited a more distinct correlation pattern. A slight positive correlation was found with age (r = 0.16), while a slight negative correlation was identified with current CD4^+^ T cell count (r = −0.06). Among routine serum biochemical parameters, IgG titers correlated positively with globulin levels (r = 0.22) and negatively with albumin levels (r = −0.09). Correlations with height (r = −0.07), duration of ART (r = 0.06), and aspartate aminotransferase (AST, r = 0.06) were negligible and not considered significant.

### 3.7. Age-Related Profiles of Anti-HAV IgG and IgM Seroprevalence

**Figure 7:**
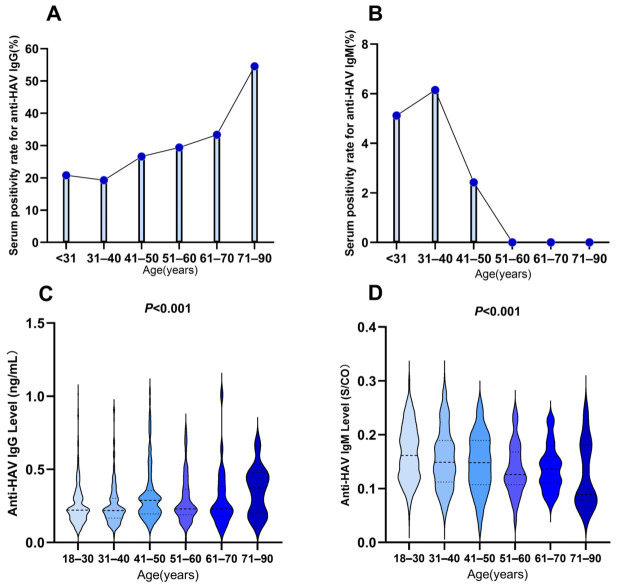
Age-associated changes in seropositivity and titers of anti-HAV IgG and IgM. (**A**) IgG seropositivity increases with age, peaking in the 71–90 years age group, (**B**) whereas IgM seropositivity peaks in the 31–40 years group before declining sharply in older age groups. (**C**) There are significant differences in anti-HAV IgG titers among age groups (*p* < 0.001). (**D**) In contrast, anti-HAV IgM titers exhibited a significant decreasing trend with age (*p* < 0.01).

Given that age has been established as a strong determinant of immunity against HAV, we observed a clear age-dependent relationship in humoral immune responses against HAV. The seroprevalence of anti-HAV IgG demonstrated a strong, positive correlation with increasing age (Figure 7A). Positivity rose progressively from 13% in participants under 31 years to 19% (31–40 years), 28% (41–50 years), 30% (51–60 years), and 33% (61–70 years), reaching a peak of 55% among individuals aged 71–90 years.

In contrast, the prevalence of anti-HAV IgM exhibited a significant inverse association with age (Figure 7B). IgM positivity was markedly higher in younger individuals and declined progressively across successive decades of life.

The quantitative relationship between antibody titers and age was further detailed. Results revealed significant differences in anti-HAV titers among the six age groups. Specifically, anti-HAV IgG titers showed an overall increasing trend with age (*p* < 0.001; Figure 7C). In contrast, anti-HAV IgM titers exhibited a decreasing trend with age (*p* < 0.001; Figure 7D).

## 4. Discussion

The data from this study demonstrate that PLWH exhibit significantly lower mean HAV IgG concentrations and seropositivity rates compared to HIV negative controls. For IgM, both antibody concentrations and seropositivity rates were comparable between groups, with no statistically significant differences observed. Multivariate logistic regression analysis revealed that HIV infection status was independently and negatively associated with IgG seropositivity (OR = 0.599, *p* = 0.007), an association that remained statistically significant after adjustment for confounders including age. Prior research demonstrates impaired immune memory in PLWH [20,21], a deficit that parallels the attenuated persistence of antibodies against other pathogens, including HBV and influenza, in this population [22,23]. This may suggest that HIV infection intrinsically constitutes a significant independent risk factor for inadequate levels of protective antibodies against HAV. These findings probably imply that due to compromised baseline immune function, PLWH have an increased susceptibility to HAV. Supporting this notion, a longitudinal study from Korea, covering 2012 to 2021, found that the age at anti-HAV IgG seroconversion gradually shifted later and the proportion of susceptible individuals increased among PLWH [24]. This pattern suggests that, without timely vaccination, HIV-infected populations may face a growing risk of HAV infection. Based on our findings, the proactive assessment of HAV immune status, achieved through quantitative anti-HAV IgG testing, may need to be integrated into the standard long-term follow-up framework for PLWH as a component of evaluating overall immunological health. Moreover, susceptible individuals living with HIV identified via screening may need to be prioritized for HAV vaccination. These proactive interventions aim to prevent individual infections and curb potential community spread.

The relatively low anti-HAV IgG seropositivity observed among HIV-negative controls (35.6%) merits attention. According to the global classification criteria proposed by Jacobsen and Wiersma, anti-HAV IgG prevalence below 50% defines a low endemicity region, characterized by a high proportion of susceptible adults and an increased risk of outbreaks [25]. Following the inclusion of the hepatitis A vaccine into China’s Expanded Program on Immunization in 2008, improvements in sanitation, hygiene, and childhood vaccination coverage have substantially reduced natural HAV exposure among younger cohorts. Consequently, many adults born during the transition era may remain susceptible to HAV infection because they neither acquired natural immunity during childhood nor received catch-up vaccination. Similar changes in HAV seroprevalence among young adults have also been reported in several regions undergoing epidemiological transition [26,27]. In contrast, highly endemic regions such as Peru still report rates of up to 98.4% [28]. Thus, the 35.6% seropositivity is appropriately considered ‘low’ both in absolute terms and in the context of epidemiological transitions, signaling an accumulation of susceptible adults and an increased risk of outbreaks. Together, these observations indicate that younger adult populations in China and in comparable settings have a low preexisting immunity to HAV, highlighting their vulnerability in the event of an outbreak.

HIV-infected individuals, particularly MSM, may have increased exposure risk to HAV due to behavioral and epidemiological factors [29]. However, anti-HAV IgG seropositivity reflects not only prior exposure, but also the ability to develop and maintain durable humoral immune responses. HIV infection is associated with immune dysregulation and impaired B-cell function, which may reduce the development, persistence, and long-term maintenance of protective anti-HAV antibodies, even in individuals with prior HAV exposure. Therefore, lower HAV IgG seropositivity in PLWH may not necessarily indicate lower HAV exposure, but rather impaired or waning antibody responses related to HIV-associated immune dysfunction.

In our study, the use of different anthropometric indicators across analyses reflects the distinct analytical objectives of the study. While BMI was applied as a standardized measure for overall between-group comparison, separate evaluation of height and weight within PLWH subgroup analyses allowed a more granular assessment of potential anthropometric associations with HAV serological status among PLWH. In multivariate logistic regression models examining IgM and IgG seropositivity, age was significantly positively correlated with anti-HAV IgG positivity and antibody titers, consistent with the classic epidemiological profile of regions with intermediate HAV endemicity, wherein cumulative exposure probability increases with age [25]. Conversely, age was inversely associated with IgM positivity; anti-HAV IgM seropositivity peaked in younger age groups, with its titer showing a negative correlation with age, a pattern consistent with established immune kinetics observed in the general population [30]. This phenomenon warrants particular vigilance, as it suggests that young PLWH may face a dynamic and under-recognized infection threat. Clinically, younger PLWH carry a dual burden: a high prevalence of IgG negativity coupled with inadequate prior exposure, or vaccination and an elevated risk of recent infection. This subgroup should therefore become a high priority for HAV vaccination.

Multivariable logistic regression was employed to determine independent determinants of HAV serostatus within the PLWH cohort. Notably, serum globulin levels were identified as an independent, strongly positive correlate of IgG seropositivity (OR = 1.099, *p* < 0.001). Meanwhile, higher CD4/CD8 ratios and baseline CD8^+^ T cell counts were associated with IgM positivity. These observations are concordant with existing clinical literature [31,32]. Together these results suggest that routine laboratory parameters, including serum globulin and the CD4/CD8 ratio, can help assess humoral immune competence and potential responsiveness to HAV antigen stimulation in clinical practice. This approach supports more precise risk stratification and individualized management.

To explore further potential determinants of anti-HAV IgM and IgG in PLWH, we examined correlations between anti-HAV antibody titers and a range of demographic, immunological, and clinical parameters. The correlation analysis revealed strikingly distinct patterns for IgM and IgG responses. Anti-HAV IgM titers showed minimal association with any of the measured variables, with all correlation coefficients falling below an absolute value of 0.2. This lack of meaningful correlation is perhaps not unexpected. According to previous research, IgM titer would be expected to be transient and primarily determined by the time since infection and the individual’s acute phase response, rather than by steady-state parameters such as current CD4^+^ T cell count or duration of ART [33]. In contrast, anti-HAV IgG titers, representing the durable memory response, exhibited a broader, albeit still modest, pattern of associations. A weak positive correlation with age (r = 0.16) was observed, consistent with the phenomenon of cumulative antigen exposure over a lifetime. More notably, a weak positive correlation was found between IgG titers and serum globulin levels (r = 0.22). As anti-HAV IgG itself constitutes a component of the gamma-globulin fraction, this association is biologically plausible. This finding underscores that in clinical decision making, reliance solely on routine parameters such as CD4^+^ T cell counts or liver function tests is insufficient for assessing HAV infection risk or vaccine-induced protection in PLWH. Instead, clinicians should use HAV-specific antibody titers alongside broader immunological and clinical data to guide HAV prevention, screening, and immunization.

Previous studies have shown that PLWH can develop humoral immune responses after vaccination and are able to achieve seroconversion [11,12,13]. However, compared with the general population, vaccine immunogenicity and the durability of protective antibody responses are often reduced in PLWH due to HIV-associated immune dysfunction [13]. Nevertheless, we still strongly recommend HAV vaccination for PLWH because vaccination remains the most effective strategy for preventing HAV infection. At the same time, considering the possibility of lower antibody titers or the shorter duration of protective immunity after vaccination in PLWH, we suggest incorporating the periodic monitoring of anti-HAV antibody titers into routine clinical follow-up to better evaluate long-term immune protection and identify individuals who may benefit from additional vaccination.

In summary, this study identifies HIV infection as an independent risk factor for diminished anti-HAV IgG levels and compromised long term protective immunity. This vulnerability is particularly pronounced among younger PLWH, who exhibit concurrently low antibody seropositivity and heightened risk of recent infection, and this concern is further underscored by the limited availability of HAV vaccinations for PLWH across regions, with a survey of 18 countries reporting availability in only 16.7% of participating countries in 2019 and 22.2% in 2022 [34]. Younger PLWH therefore belong to a priority group for HAV vaccination and routine serological follow-up. In clinical practice, assessments of HAV infection risk or vaccine efficacy should not rest solely on conventional immune markers such as CD4^+^ T cell counts or transaminase levels. Rather, such evaluations must integrate HAV-specific antibody testing and consider markers reflective of immune reserve (e.g., serum globulin) to enable accurate risk stratification and individualized immunization management. Future investigations should prioritize age matched designs to more precisely delineate the authentic landscape of HAV immune responses in PLWH.

However, this study has several limitations. First, its cross-sectional design precludes causal inference and limits the assessment of dynamic changes in immune memory over time. Second, the control group had less comprehensive clinical data, including detailed serological profiles, behavioral risk factors, and longitudinal immunological parameters, compared with the PLWH cohort. This restricted our ability to further explore determinants in control groups. Additionally, HAV vaccination history was self-reported and may have been affected by recall bias. Several methodological limitations should also be acknowledged. First, the HIV-negative control group was recruited from the Health Management Center, whereas PLWH were enrolled from the Department of Infectious Diseases. Although this real-world sampling strategy improved representativeness, it may have introduced selection bias and limited strict comparability between groups. Second, while multivariable adjustment was performed, more comprehensive statistical diagnostics, including formal collinearity assessment and additional model robustness analyses, were not systematically conducted. Therefore, these findings should be interpreted cautiously and considered exploratory. Future prospective multicenter studies with more rigorously matched control populations and enhanced statistical validation are warranted to confirm the generalizability of our results. Despite these constraints, the findings provide valuable epidemiological evidence to guide HAV prevention strategies in PLWH.

## 5. Conclusions

This study identifies HIV infection as an independent risk factor for lower anti-HAV IgG seropositivity and antibody titers compared with HIV-negative individuals. Within the PLWH cohort, age and serum globulin levels emerged as independent factors of anti-HAV IgG seropositivity, whereas age, baseline CD8^+^ T cell count, CD4/CD8 ratio, and lymphocyte count were independently associated with anti-HAV IgM seropositivity. These findings support incorporating routine anti-HAV IgG screening into the long-term care of all PLWH, alongside the targeted vaccination of susceptible individuals to reduce both personal infection risk and potential community transmission. Younger PLWH require particular attention due to their concurrent low seropositivity and ongoing HAV exposure.

## Figures and Tables

**Table 1:** Demographic characteristics of PLWH group and Control group.

Characteristic	Control Group	PLWH Group	*p* Value
Sample size, *n*	432	800	-
Sex			<0.001
Male	222 (51.4%)	731 (91.4%)	
Female	210 (48.6%)	69 (8.6%)	
Ages, years	34.94 ± 11.51	35.94 ± 11.90	0.151
BMI	21.88 ± 3.73	21.64 ± 4.06	0.457
Anti-HAV IgM			0.212
Positive	29 (6.7%)	40 (5.0%)	
Negative	403 (93.3%)	760 (95.0%)	
Anti-HAV IgG			<0.001
Positive	154 (35.6%)	180 (22.5%)	
Negative	278 (64.4%)	620 (77.5%)	

PLWH, People living with HIV; BMI, Body Mass Index; HAV, Hepatitis A Virus.

**Table 2:** Association of HAV IgM Status and Clinical variables in PLWH Group.

	PLWH Group		
Characteristic	HAV IgM (−)	HAV IgM (+)	*p* Value
Sample size, *n*	760	40	—
Sex			0.370
Male	696 (91.6%)	35 (87.5%)	
Female	64 (8.4%)	5 (12.5%)	
Ages, years	36.19 ± 12.06	31.23 ± 6.84	<0.001
Height	168.33 ± 7.53	169.18 ± 7.63	0.489
Weight	61.48 ± 12.95	60.57 ± 10.66	0.669
Transmission route			0.761
MSM	632 (83.2%)	34 (85%)	
Non-MSM	128 (16.8%)	6 (15%)	
Duration of ART (years)	2.71 ± 1.95	2.90 ± 1.70	0.501
Marital Status			0.256
Married	200 (26.3%)	9 (22.5%)	
Single	491 (64.6%)	30 (75.0%)	
Divorced	69 (9.1%)	1 (2.5%)	
Co-infected with Syphilis			0.911
Negative	564 (74.2%)	30 (75.0%)	
Positive	196 (25.8%)	10 (25.0%)	
Co-infected with HBV			0.429
Negative	695 (91.5%)	38 (95.0%)	
Positive	65 (8.5%)	2 (5.0)	
CD4^+^ T cell at baseline	297.15 + 183.61	326.51 + 191.30	0.367
CD4^+^ T cell	511.89 + 250.58	580.45 + 285.76	0.144
CD8^+^ T cell at baseline	1036.28 + 684.13	1135.97 + 1097.53	0.593
CD8^+^ T cell	853.39 + 681.83	760.98 + 658.21	0.392
CD4/CD8^+^ T cell ratio at baseline	0.34 + 0.29	0.408 + 0.30	0.201
CD4/CD8^+^ T cell	0.77 + 0.90	0.96 + 0.52	0.030
White blood cell (10^9^/L)	6.49 + 3.16	6.21 + 1.74	0.971
Lymphocyte (10^9^/L)	2.18 + 0.72	2.08 + 0.68	0.393
Platelet (10^9^/L)	246.46 + 61.97	247.53 + 56.21	0.908
Platelet Distribution Width (%)	11.20 + 1.95	11.19 + 1.57	0.964
Alanine Aminotransferase (U/L)	29.79 + 28.44	25.55 + 13.17	0.111
Aspartate Aminotransferase (U/L)	25.68 + 19.10	22.16 + 7.20	0.025
Albumin (g/L)	47.76 + 17.49	47.10 + 2.26	0.364
Globulin (g/L)	27.95 + 5.73	27.79 + 3.67	0.801
Total Bilirubin (μmol/L)	10.69 + 0.42	3.87 + 0.70	0.041
Direct Bilirubin (μmol/L)	3.65 + 2.01	3.33 + 1.27	0.190

**Table 3:** Association of HAV IgG Status and Clinical variables in PLWH Group.

	PLWH Group		
Characteristic	HAV IgG (−)	HAV IgG (+)	*p* Value
Sample size, *n*	620	180	—
Sex			0.051
Male	573 (92.4%)	158 (87.8%)	
Female	47 (7.6%)	22 (12.2%)	
Ages, years	35.20 ± 11.27	38.49 ± 13.58	0.003
Height	168.62 ± 7.38	167.51 ± 8.03	0.117
Weight	61.43 ± 12.91	61.41 ± 12.63	0.592
Transmission route			0.045
MSM	525 (84.7%)	141 (78.3%)	
Non-MSM	95 (15.3%)	39 (21.7%)	
Duration of ART (years)	2.75 ± 1.95	2.62 ± 1.90	0.412
Marital Status			0.178
Married	156 (25.2%)	53 (29.4%)	
Single	414 (66.8)	107 (59.4)	
Divorced	50 (8.0%)	20 (11.2%)	
Co-infected with Syphilis			0.649
Negative	458 (67.4%)	136 (75.6%)	
Positive	162 (32.6%)	44 (34.4%)	
Co-infected with HBV			0.982
Negative	568 (91.6%)	165 (91.7%)	
Positive	52 (9.4%)	15 (8.3%)	
CD4^+^ T cell at baseline	298.68 + 178.32	298.47 + 202.75	0.990
CD4^+^ T cell	522.84 + 252.55	489.31 ± 252.21	0.118
CD8^+^ T cell at baseline	1083.46 + 934.65	1128.78 + 628.62	0.480
CD8^+^ T cell	854.56 + 677.59	828.81 ± 692.25	0.659
CD4/CD8^+^ T cell ratio at baseline	0.34 ± 0.27	0.36 ± 0.38	0.693
CD4/CD8^+^ T cell	0.78 ± 0.97	0.75 ± 0.50	0.611
Lymphocyte (10^9^/L)	2.19 ± 0.71	2.12 ± 0.74	0.226
White blood cell (10^9^/L)	6.57 ± 3.12	6.36 ± 1.73	0.377
Platelet (10^9^/L)	247.70 ± 61.77	242.090 ± 61.27	0.273
Platelet Distribution Width (%)	11.21 ± 1.85	11.18 ± 2.19	0.889
Alanine Aminotransferase (U/L)	29.23 ± 27.63	30.92 ± 29.04	0.518
Aspartate Aminotransferase (U/L)	25.07 ± 18.55	27.10 ± 19.40	0.250
Albumin (g/L)	48.04 ± 19.23	46.64 ± 3.13	0.084
Globulin (g/L)	27.34 ± 4.57	30.01 ± 8.04	<0.001
Total Bilirubin (μmol/L)	8.81 ± 5.56	8.25 ± 4.99	0.232
Direct Bilirubin (μmol/L)	3.67 ± 2.08	3.50 ± 1.61	0.346

## Data Availability

Authors can confirm all relevant data are included in the article and materials are available on reasonable request from the authors.

## Abbreviations

The following abbreviations are used in this manuscript:

PLWH	People living with HIV
HAV	Hepatitis A Virus
HBV	Hepatitis B Virus
HCV	Hepatitis C Virus
IgM	Immunoglobin M
IgG	Immunoglobin G
BMI	Body Mass Index
MSM	Men who Have Sex with Men
ART	Duration of Antiretroviral Therapy
WBC	White Blood Cell Count
PLT	Platelets
PDW	Platelet Distribution Width
LYM	Lymphocyte Count
ALB	Albumin
BL-CD4^+^ T cell	CD4^+^ T cell at baseline
BL-CD8^+^ T cell	CD8^+^ T cell at baseline
BL CD4/CD8 ratio	CD4^+^ T cell/CD8^+^ T cell at baseline
ALT	Alanine Aminotransferase
AST	Aspartate Aminotransferase
TB	Total Bilirubin
DB	Direct Bilirubin
